# Investigations on Flexural and Compressive Strengths of Mortar Dedicated to Clinker Units—Influence of Mixing Water Content and Curing Time

**DOI:** 10.3390/ma15010347

**Published:** 2022-01-04

**Authors:** Jan Kubica, Iwona Galman

**Affiliations:** Department of Structural Engineering, Silesian University of Technology, 44-100 Gliwice, Poland; jan.kubica@polsl.pl

**Keywords:** mortar mixing water content, mortar flexural strength, mortar compressive strength, clinker units, curing time, three-point bending test

## Abstract

The article presents laboratory tests on the impact of the mixing water content used in the preparation of fresh mortar on the flexural and compressive strength of one of the dry-mix mortars produced by a leading European producer and dedicated to bricklaying with clinker elements. The development of these parameters in relation to curing time was also analyzed. The mortar samples were prepared from a factory-made mortar mix using 4.0 L (the value recommended by the mortar manufacturer), 4.5 L, and 5 L of water per 25 kg bag of ready-made, pre-mixed dry mortar mix. All samples were tested in five series after 5, 9, 14, 21, and 28 days of sample curing. The results of these tests showed that the use of 6 and 18% more mixing water than recommended by the manufacturer (4.5 and 5 L per bag) adversely affected the basic mechanical parameters of the tested mortar. Moreover, it was found that the highest compressive strength values were obtained after 21 days of curing and not after 28 days as usual. It was also found that hardening time and higher than recommended water content adversely affected the bending strength of the mortar.

## 1. Introduction

Not only the correct selection of the mortar for a specific type of masonry but also its proper preparation has a significant impact on the behavior of a structural element or even the entire structure. It is very important to ensure that the mortar has both appropriate mechanical parameters (compressive and bending strength) and appropriate workability for a period of time that allows its wear, especially in periods of elevated external temperatures (e.g., in summer) or when it is necessary to make prefabricated façade wall panels. This usually requires the use of more mixing water than recommended. This is especially important in the case of more and more frequent use of ready-made dry mortar mixes, where the producers of these mortars specify the minimum and maximum amount of water that should be used to obtain the declared mechanical parameters and adequate durability. The second problem that arises both when using mortars for bricklaying the facing leaflets of external enclosure walls of buildings, and when making prefabricated wall panels, is the need to ensure a relatively quick increase in the mechanical parameters of the masonry, which allows for faster erecting of masonry structures. It is connected with shorter time of façade execution, which has an impact on costs.

It is commonly assumed that increasing the amount of mixing water, which of course also increases the water-binder ratio, leads to deterioration of the mechanical parameters of the mortar, including the values of flexural and compressive strengths, as well as bond strength of masonry. However, in the light of the available research results, this is not always true. In some cases, an increase in water content may ultimately result in an increase in bond strength of masonry, which is a key parameter influencing the mechanical parameters of the masonry. As a result, the mechanical parameters are also improved, not only of the masonry but also of the mortar. The positive effect of a higher proportion of mixed water can be related to many different parameters. These parameters include the unit absorption characteristics (higher water absorption of masonry units may cause drainage of a significant amount of excess water from the fresh mortar), initial absorption rate (IRA), sorptivity, flow and retention, curing time and conditions, and performance quality [1,2]. Two of the above parameters seem to be of particular importance, namely, IRA and water absorption [3,4,5,6,7]. All the above parameters are directly or indirectly related to the water content during the preparation of the mortar mixture. According to the research results on the influence of the amount of mixing water on the mechanical parameters of lime mortars [5] and the bond strength of brick walls into hydraulic lime mortar [8], higher values of the respective strengths were obtained for higher water content. One should also not forget about the very significant influence of workability on the mechanical parameters of mortar and masonry [9], especially masonry based on lime mortar [10].

It is worth bearing in mind that not the initial water content, but rather water retention and absorption of masonry units, has a much greater impact on the mechanical parameters of the mortar and the bond strength (flexural strength of the masonry). The main reason is the ability to maintain moisture under appropriate hardening conditions and to maintain appropriate plasticity of fresh mortar. At the same time, it should be remembered that the higher water content in the fresh mortar might in some cases reduce its mechanical parameters, and in other cases even increase them [11,12,13,14]. A similar effect was also observed with regard to the tensile strength of the cement mortar [15]. With an increase in the mortar saturation with water, the tensile strength, maximal deformation, and fracture toughness, the cement mortar samples first showed a decrease and then an increase. The amount of mixing water in the mortar is also related to shrinkage, especially during the hardening and drying phase. As a result, it also negatively affects the strength of the mortar, and thus the bond strength of the masonry (related to the adhesion between the mortar and the masonry elements). The research in [16] showed that the strength of the material has a greater influence on the bond strength than the roughness of the masonry elements and the bond strength is related to the material shrinkage.

The influence of curing and hardening conditions, as another factor influencing the mechanical parameters of the mortar and the bond strength of the mortar to the masonry elements, has already been analyzed experimentally. The results of the research [17,18] show that the correct hardening process has a positive effect on the increase of adhesion, and thus the bond strength value; however, the scale of growth depends at the same time on the presence of various additives, such as plasticizers, which significantly delay the setting time. One such natural plasticizer is, of course, lime.

Moreover, the proper hardening conditions consistently determine the shrinkage value, mainly the initial (early age) shrinkage value, and its influence on the bonding effect in the mortar, and in the case of masonry, on the bond strength value (adhesion between the mortar and the bed surface of masonry units). However, since the important phenomenon of drying, shrinkage is more directly related to curing time and seasoning conditions. It was this aspect that was the second factor, besides water content, taken into account in this study.

The examples described above clearly show that a higher water content and thus a higher water–binder ratio can in some cases have a positive effect on the mechanical parameters of the hardened mortar. On the other hand, larger amounts of water are usually responsible for the increase in porosity, also quite often negatively affecting the bond strength, which was presented in research [19]. In addition, in the case of masonry units characterized by low water absorption, which are usually masonry made of clinker units, a layer of water may form on the contact surface of the two components of the wall, mortar, and masonry elements, reducing adhesion, and thus reducing the bond strength. Therefore, in the presented research, attempts were made to analyze the effect of both the amount of mixing water and the curing time and conditions of mortar on its basic mechanical parameters, namely, flexural and compressive strength.

## 2. Motivation, Material, Program, and Technique

Conducting the presented research was dictated by the problems of a construction company preparing to make a clinker façade of a public utility building. The outer façade wall was designed in the form of prefabricated wall panels made of clinker elements with the use of the recommended system mortar. The technological process of making prefabricated panels required the use of more mixing water than recommended by the manufacturer of the mortar. At the same time, the manufacturer’s technical department informed that increasing the mixing water by even 20% should not adversely affect the mechanical parameters of the mortar. The contractor had reservations about these recommendations, and therefore it was necessary to carry out of the presented experimental investigations. The result of the presented tests should allow for changes in the technological process of prefabrication of façade panels so that they meet the requirements of load capacity and safety of use.

In the presented research, a ready-made mortar (premixed dry mix) of one of the leading European producers was used. According to the manufacturer’s declaration, the mortar is classified in terms of strength as M10, meeting the requirements of EN 998-2:2016-12 [20]. Unfortunately, the composition of the mortar is a secret of the manufacturer and is not presented or available. The information that the authors were able to obtain shows that the main component of the binder is obviously Portland cement, but it is not known whether it is pure clinker cement (CEM I) or cement with the addition of ash or slag (CEM II, CEM IV, or CEM V—according to EN 197-1: 2002 [21]). The binder also contains calcium hydroxide and an air-entraining additive, a plasticizer, and sealing additives (no name given). The producer of the mortar mix does not specify the content of cement and calcium hydroxide in the composition of the binder. It only states that the content of calcium hydroxide does not exceed 5% of the dry weight of the mixture. The content of crystalline silica (sand) is not less than 75% of the dry weight of the total mixture. The manufacturer of the mortar does not state whether the sand meets the requirements of EN 13139: 2002 [22], but only that it ensures that the appropriate dense structure of the hardened mortar is obtained. Additionally, there is information in the description of the mortar that it contains the addition of Rhine trass and is dedicated to bricklaying and pointing bricks with water absorption up to 10%. Therefore, it is dedicated to the bricklaying and pointing of masonry made of bricks and clinker fittings, the absorption of which usually does not exceed 6%. According to the manufacturer’s information, the prepared mortar should be used within 60 to 90 min.

These material tests of the mortar were performed in order to determine the effect of the amount of mixing water used on the values of basic mechanical parameters, i.e., compressive strength and flexural strength. The second variable parameter was the curing time of the samples. The samples were tested after 5, 9, 14, 21, and 28 days of curing. The research used a mortar specially dedicated to clinker masonry units in the form of a dry mix, to which only the appropriate amount of water should be added. In the case of the mortar used, the manufacturer recommends using 3.75–4.25 L per 25 kg of the dry mix. After conversion, it can be assumed that the manufacturer recommends the use of mixing water in an amount of 15% to 17% of the dry mix weight. In total, the research covered three basic series, differing in the content of mixing water. In the case of the first batch, the given water content was in line with the manufacturer’s recommendations (4 L of water for 25 kg of dry mix). In the second batch, 4.5 L of water per 25 kg of dry mix was used. The third batch consisted of samples made with 5 L of water for each 25 kg of dry mix. The water amount exceeded the maximum recommended value by the mortar producer by over 17.5%. Tap water was used to prepare the mortar, because on the construction site, in practice, such water is used in all construction works. Of course, it is not chemically pure water, because it is usually characterized by a low carbohydrate hardness and is slightly chlorinated for sanitary reasons.

Both the flexural and compressive strength tests of the hardened mortar were carried out in accordance with EN 1015-11: 1999 [23]. On the basis of information from the mortar manufacturer, the preparation and conditioning of storing specimens were performed as for mortar with hydraulic binder where cement and air-lime not exceeding 50% of the total binder mass. Specimens, removed from the polyethylene bag, were stored under controlled conditions (in humidity of 65% ± 7% and at the temperature of 20 °C (±2 °C) in the laboratory in a climate chamber. The test program is presented in Table 1.

**Figure 1:**
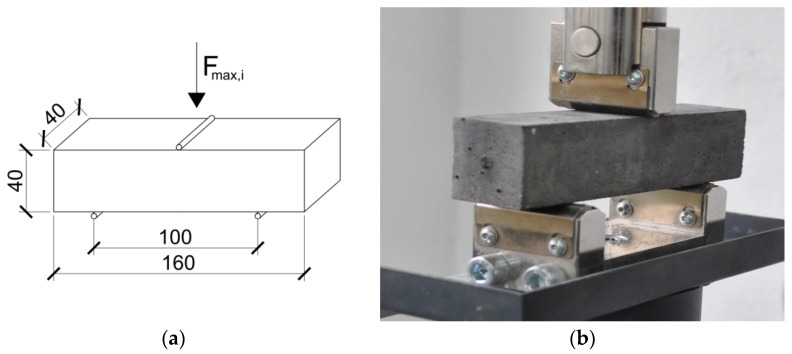
Determination of flexural strength: (**a**) dimensions of the beam-specimens and static scheme; (**b**) the view of the specimens in testing machine ready to test.

The determination of the bending strength was carried out on standard beam specimens with dimensions as shown in Figure 1a. The samples were subjected to a three-point bending test, loaded with a single concentrated force applied in the center of the span, increasing uniformly until the moment of failure. The distance between the points of support was 100 mm, and the total length of the beam specimen was 160 mm. The static diagram applied loads and the geometry of the tested elements are shown in Figure 1a, while Figure 1b shows the research element prepared for testing in the controls machine—model no. 65-L27C12. The load on the samples was applied at a uniform rate of the order of 15 N/s. 

**Figure 2:**
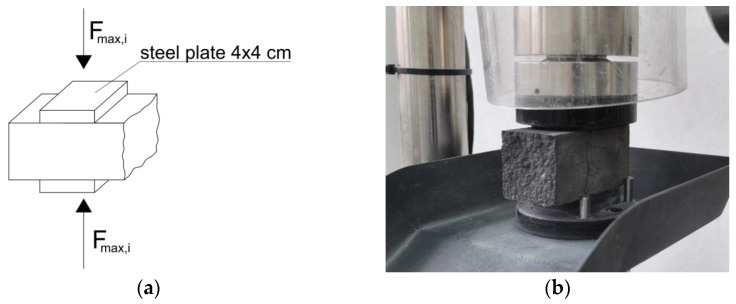
Determination of compression strength: (**a**) static scheme, loading, and geometry of tested specimens; (**b**) the view of the specimen ready to test.

In accordance with the provisions of EN 1015-11: 1999 [23], we determined the compressive strength of the mortar on the beam halves obtained from bending tests. The obtained test pieces of the mortar were subjected to axial compression through steel plates of a square shape and dimensions of 4 × 4 cm. The static scheme, load, and geometry of the compressed half-beam elements are shown in Figure 2a. In turn, Figure 2b shows the view of the test specimen, ready to test.

## 3. Mechanism of Cracking Failure

**Figure 3:**
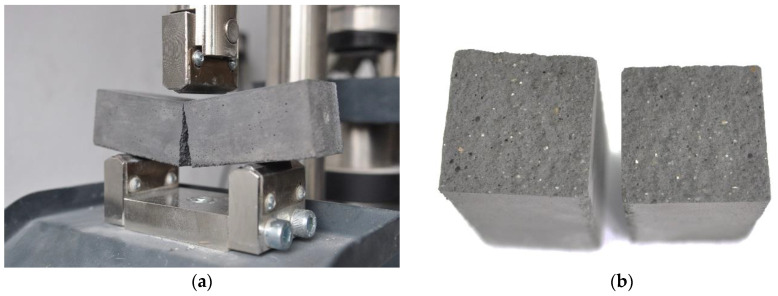
Mode of failure of the beam specimen: (**a**) flexural crack; (**b**) fracture view of the cracked sample.

The failure mechanisms of the three-point bending beam elements were identical in all the tests performed. The mode of cracking always was through a vertical scratch, regardless of the curing time or the water content used to prepare the mortar. Selected sample photos, showing a cracked element and a view of its fracture, are shown in Figure 3. In the presented research, no clearly visible differences were found in the appearance of the fracture plane of the examined trabeculae. All mortar beams in the fracture plane had a large number of pores visible, as can be seen in Figure 3b. This is, of course, because the manufacturer declares this mortar frost-resistant. A significant number of pores is, on the one hand, the use of trass, which, due to its physical structure, is a component that naturally and physically aerates the mixture; on the other hand, it results from the using of an air-entraining additive. Unfortunately, as previously stated, the manufacturer did not provide the chemical composition of the mortar to the authors. It should also be borne in mind that air-entraining additives improve workability and frost resistance of the mortar but lower the strength parameters and its adhesion to masonry elements. The high porosity of the mortar ensures its frost resistance and allows for the accumulation of efflorescence and salting (i.e., in fact alkaline compounds) and not allowing them to go outside, which is particularly important in façade walls.

**Figure 4:**
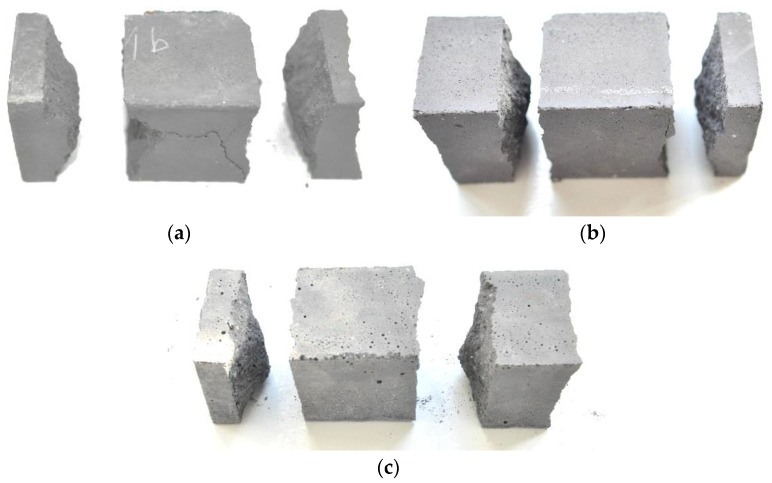
Examples of failure after the compression test of semi-beam samples made with the use of: (**a**) 4 L of mixing water; (**b**) 4.5 L of water; (**c**) 5 L of water.

Similarly, in the case of determining the compressive strength of the mortar on the halves of the beam specimens previously subjected to three-point bending, no significant differences were found in the failure mechanisms depending on the hardening time and the mixing water content used for the mortar mix preparation. All the tested samples were damaged in an almost identical, typical way. Examples of photos of failure after the compression test of semi-beam samples made with the use of different amount of mixing water are shown in Figure 4. It can be seen that the increase in the amount of mixing water caused a slight but visible increase in the porosity of the samples. Especially when using 5 L of mixing water, a large number of pores are visible on the sample surfaces. The measured diameters of the largest pores exceeded 1 mm.

In order to explain the reasons for the visible changes in the porosity of the mortar depending on the amount of mixing water used, we must briefly explain what chemical reactions and physical processes occur in it. First, the matter of the cement used in the mortar. Although no information is available on the type of cement, it can be presumed that the used Portland cement with additives (fly ash and/or slag), the main ingredient of which is Portland clinker produced by sintering raw materials containing CaO, SiO_2_, MgO, FeO, and small amounts of other materials. The weight ratio (CaO)/(SiO_2_) should not be less than 2, and the MgO content should not exceed 5.0% [21]. The hydraulic additive is usually granular slag, which has latent hydraulic properties that can only react and harden with appropriate activation. Portland cement may also contain the addition of fly ash, which has hydraulic and/or pozzolanic properties, which causes it to react with calcium hydroxide in the aqueous environment, resulting in the C–S–H phase because of the reaction [24]. 

Chemical activity in cement-water and lime-water systems (pozzolanic reaction) lead to the formation of hydrated calcium silicates xCaO · SiO_2_ * (x + y) H_2_O (C-S-H) and hydrated calcium silicates and aluminates xCaO · SiO_2_ · Al_2_O_3_ · (x + y) H_2_O (C-S-A-H) according to the following, well known, reactions:

SiO_2_ + xCa(OH)_2_ + yH_2_O → xCaO · SiO_2_ · (x + y) H_2_O (1)

SiO_2_ + Al_2_O_3_ + xCa(OH)_2_ + yH_2_O → xCaO · SiO_2_ · Al_2_O_3_ · (x + y) H_2_O (2)

When pozzolanic additives are introduced into the cement, the total content of calcium hydroxide is lowered, while additional amounts of hydrated calcium silicates are formed in its place. The C–S–H (and C–S–A–H) phases fill pores of large diameters in cement slurries, changing the share of particular types of pores in the hardened cement paste. The content of capillary pores, which are dangerous from the point of view of the strength and durability of mortars, is reduced in favor of gel pores with a very small diameter. As a result, the structure of the hardened material becomes more compact, creating a material with increased strength and durability. The pozzolanic reaction is a relatively slow reaction, and positive effects should be expected after longer maturation times of mortar. Therefore, to shorten this time, additives are added to the binder composition to accelerate bonding and hardening of the mortar. The use of a larger amount of make-up water than it results from the chemical binding of the binder components means that some of the water molecules cannot react and be permanently bound. As a result, during the process of setting and hardening the mortar, part of this water evaporates (due to the heat of hydration) and is retained in the lattice skeleton, which is manifested by an increased number of pores, also visible on the surface of the samples on the surfaces in contact with the mold elements. The effect of the additive in the form of fly ash on the amount of spontaneous shrinkage, micro crack formation, and fracture toughness is known. For example, it was also analyzed in relation to lightweight aggregate concretes [25].

## 4. Test Results and Discussion

The basic results of the presented experimental investigations for all tested series are shown in tabulated form in Table 2, Table 3 and Table 4. In these tables the following method of marking individual types of test elements has been adopted: mFT—beam specimens subjected to three-point bending test; mCT—specimens in form of half of beams taken from bending test; 0.5 to 28—days of curing before testing; 4.0, 4.5, or 5.0—water amount (in liters) used per one 25 kg bag of factory-ready dry mortar mix. In the case of the results of the three-point bending tests, due to the fact that three beam specimens were tested each time, only the average values of both the maximum loading force F_av_ and the flexural strength f_mx,av_ were determined. However, in the case of determining the compressive strength of the mortar on the halves of the beams taken from bending tests, apart from the average values of the maximum compressive force F_av_ and the compressive strength f_c,av_, the values of coefficient of variation (CoV) ν**_L_** were also calculated and presented.

The flexural strength of the test specimens as it was expected increases with the curing time. However, this is observed only in the case of samples made of mortar, for the preparation of which 4 L of water were used per 25 kg of dry mix (the average value in the range from 3.75 to 4.25 L recommended by the producer of this mortar). In this case, the flexural strength after 14 days was practically the same as after 28 days. The greatest increase of flexural strength occurred between the 9th and 14th day of curing. This was because the manufacturer has optimized the composition of the binder with the amount of water needed for the setting and hardening of the mortar. This means that there should be no significant amount of unbound water in the crystal lattice of the hardened mortar. The structure of the hardened mortar reached the appropriate density, which in turn guaranteed the appropriate mechanical parameters. It is a generally known principle of the correct selection of the binder composition in mortars.

In case of elements made with a higher water content, a clear increase in strength occurred only between the 14th and 21th day of curing. On the other hand, a slight decrease in strength was noted on samples tested after 28 days. This applies to both the mortar, prepared with the use of 4.5 L, and 5 L of mixing water for every 25 kg of the dry mix. This phenomenon can be explained by the fact that not all of the water molecules are involved in the chemical reactions. This causes an extension of the bonding process time and the formation of the crystal lattice, an increase in shrinkage, especially in the first days of hardening, due to the excess of water particles, which did not take part in the chemical bonding process and must partially evaporate, and partially become trapped in the crystal lattice, causing its weakening. The more excess water, the greater its net effect on the hardening of the mortar and its mechanical parameters. Finally, the higher water content changes the curing conditions of the sample and contributed to higher shrinkage. Mainly because of the shrinkage, during the drying mortar process, can be seen as the formation of micro cracks, which ultimately results in a reduction in its mechanical parameters.

**Figure 5:**
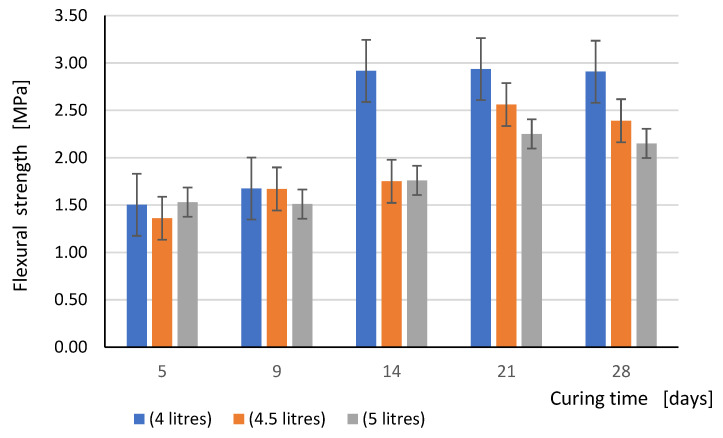
Flexural strength in dependence with the curing time (days) for all tested series.

**Figure 6:**
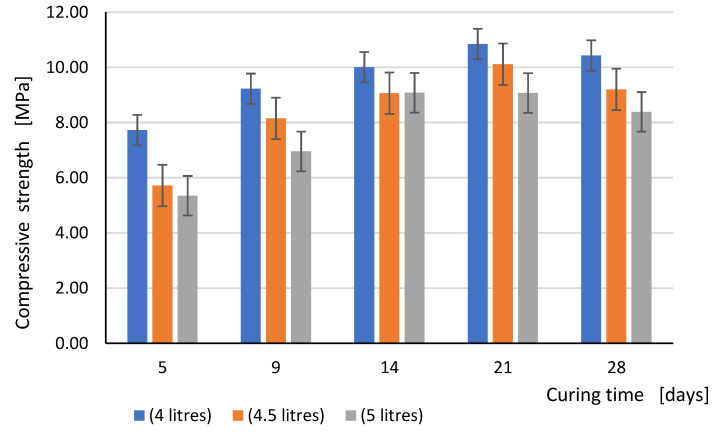
Compressive strength in dependence with the curing time (days) for all tested series.

The comparison of the flexural strength and compressive strength test results obtained for all three values of the mixing water content in relation to curing time is shown graphically in the form of bar graphs in Figure 5 and Figure 6, respectively.

The presented research confirmed that the age of the test specimens at the time of the test has a significant impact on the compressive strength of the mortar (similar also to flexural strength). In the case of samples from the mortar for which 4 and 4.5 L of water were used for 25 kg of dry mix, an almost linear increase was observed between the strength values corresponding to the tested samples between the 5th and 21th day of curing, while after 28 days, the compressive strength decreased slightly. The situation is slightly different with regard to the samples from the mortar, in which the greatest amount of water was used, i.e., 5 L. An almost linear increase in compressive strength occurred between the 5th and 14th day of curing. Then, the compressive strength started to decrease until after 28 days it reached the level of about a little more than 90% of the maximum value (obtained after 14 days). The decrease of compressive strength after 28 days of curing, as mentioned before, was probably due to the formation of shrinkage micro cracks, which had not yet resulted in visible cracks, but had already reduced the tensile strength of the mortar and consequently also the compressive strength. 

In analyzing the dependencies shown in Figure 6, we noticed that for each curing period, the compressive strength determined on samples made of mortar with 4.5 L of water per 25 kg of dry mix was on average about 15% higher than in the case of samples made of more water (5 L) and 15% smaller in comparison to the sample prepared according to the manufacturer’s recommendations (4 L). Thus, the amount of water used to prepare the mortar had a significant impact on the compressive strength of the samples, regardless of the curing time. When analyzing the values of compressive strength after 28 curing days, we found that the average value of the compressive strength slightly exceeded 10 MPa, and therefore it was in line with the strength class M10 declared by the mortar manufacturer. On the other hand, the use of a larger amount of mixing water resulted in a decrease in strength after 28 days to the values of 9.2 MPa (for 4.5 L) and 8.39 (for 5 L of water).

**Figure 7:**
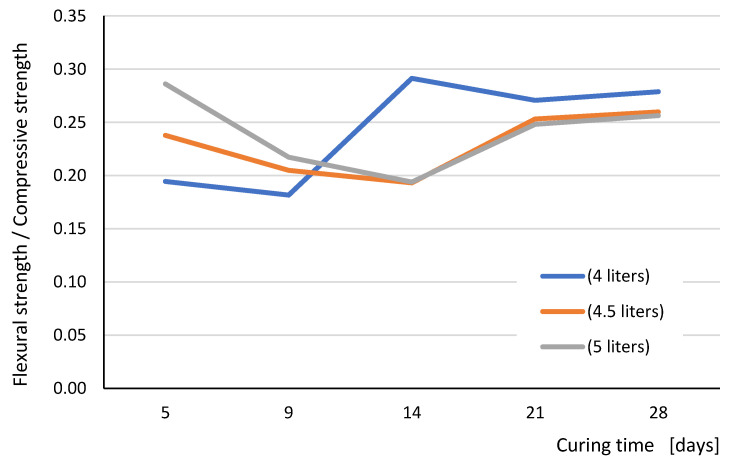
The flexural strength—compressive strength relationships in dependence with the curing time (days) for all tested series.

In turn, Figure 7 graphically shows the relationship of the ratio of flexural strength to compressive strength as a function of curing time. It can be seen that for the mortar for which more water was used than recommended by the manufacturer (4.5 L and 5 L), this ratio was the same for individual curing periods. Only in the case of mortar made with the use of 4 L (in accordance with the recommendations of the mortar manufacturer), from the 14th day of curing, was this ratio almost constant. It proves more optimal conditions of setting and hardening of the mortar.

For the recommended amount of mixing water, the flexural strength increases to the level corresponding to the maximum value. This is because the manufacturer has optimized the composition of the binder with the amount of water needed for the setting and hardening of the mortar. This means that there should be no significant amount of unbound water in the crystal lattice of the hardened mortar. The structure of the hardened mortar reached the appropriate density, which in turn guaranteed the appropriate mechanical parameters. For the other two amounts of mixing water, not all of the water was involved in the chemical reactions. This caused an extension of the bonding process time and the formation of the crystal lattice, an increase in shrinkage, especially in the first days of hardening, due to the excess of water particles, which did not take part in the chemical bonding process and must partially evaporate, and partially become trapped in the crystal lattice, causing its weakening. The more excess water, the greater its negative effect on the hardening of the mortar and its mechanical parameters.

In the case concerning a mortar made with the recommended amount of mixing water, i.e., 4 L per 25 kg bag, the formation of shrinkage micro cracks inside the test pieces mortar influenced, although to a much lesser extent, the flexural strength also. The average value of the flexural strength after 21 days of curing was 2.94 MPa, and after 28 days, it was 2.91 MPa (decrease by approximately 1%). However, in the case of the compressive strength, these values were, respectively, 10.84 MPa and 10.43 MPa (decrease by approximately 4%). According to the authors, the fact that micro cracks did not affect the flexural and compressive strength in the same way can be explained by the trajectory of the principal tensile stresses in these two cases. The contraction micro cracks are created in the mortar in different directions. When bending, cracks oriented perpendicular to the trajectory of the principal tensile stresses (horizontal direction in bending) have a negative effect. On the other hand, in compression, tensile stresses act in the entire horizontal plane (perpendicular to the applied load), and compressive stresses in the direction of the acting load (vertical). Therefore, both mainly horizontally and vertically oriented micro cracks have a negative impact. Of course, the above also applies to the components of all micro cracks with directions oblique to the main directions. It should also be remembered that when bending in the middle section, there is a completely different distribution of tensile stresses than in the horizontal section of compression samples. This also has a negative effect on the micro cracks in different areas of the sample. As a result, the influence of the micro cracks on the bending strength is slightly smaller than on the compressive strength. In the other two cases, more unbounded water has a more pronounced negative effect on both flexural and compressive strength.

By analyzing the relationships shown in Figure 7, we notice that the ratio of bending strength to compressive strength ranges from about 0.2 in the first two weeks of curing to 0.28 after 28 days of curing for the mortar made with the recommended amount of 4 L of water and 0.26 for both cases of using more mixing water. It can be assumed for a mortar made with the use of the amount of mixing water recommended by the manufacturer that for a 28 day strength, the flexural strength (f_mx_) for analyzed mortar can be calculated on the basis of the value of compressive strength (f_c_) from the formula:

(3) $$f_{\mathrm{mx}}=0.9\;\cdot \;\sqrt{f_{c}}$$

For larger values of the mixing water content, the coefficient before the elemental expression in this formula should be lower. For the presented studies, it is in the range from 0.8 to 0.75.

A similar value, of the order of 0.29, was obtained for 28 days of curing in sand concrete studies, with a composition similar to the presented mortar [26]. For similar cement mortars, the values of the flexural strength/compressive strength ratio obtained in the Arab research for the ready mixes produced on the local market oscillated between 0.33 and 0.38 [27]. On the other hand, tests of modified mortars with ethylene-vinyl acetate (EVA) gave a lower strength ratio of 0.25 [28].

## 5. Conclusions

The paper presents the results of experimental research on the effect of the amount of mixing water used to prepare the mortar and the hardening time on its basic mechanical parameters, i.e., bending and compressive strength for one of the mortars for masonry made of clinker elements widely available on the building market. Contrary to the manufacturer’s assurances, the tested mortar turned out to be very sensitive to changes of the mixing water content. The increase in the amount of mixing water by about 6% and 17.5% in relation to the maximum value recommended by the manufacturer, i.e., 4.25 L per 25 kg of dry mix, had a negative effect on both tested mechanical parameters of the mortar. 

As expected, the best parameters were demonstrated by the mortar, which was prepared with the amount of water recommended by the manufacturer. After 14 days of curing, the mortar achieved almost the maximum values of both parameters. The properties of mortar samples to which more water was used were slightly different, as is usually necessary when carrying out bricklaying works in periods of high temperatures (summer period) or in the prefabrication of façade panels, when it is necessary to ensure higher workability of the mortar. Increasing the amount of mixing water resulted in a significant decrease in both the compressive strength and the flexural strength. The decrease in the values of flexural strength after 28 days were 18% (for 6% more mixing water) and 26% (for 18% more water), respectively, and the compressive strengths were lower at 12% and 20%, respectively. This means that the addition of water by about 6% resulted in the fact that after 28 days, the mortar did not reach the strength for the assumed strength class M10.

When analyzing the behavior of the mortar during the entire 28 days of curing, we observed that the maximum values of the mortar flexural and compressive strength were obtained for 21 days of seasoning. The samples, especially from the mortar with more mixing water, tested after 28 days of curing had a slightly lower value, which was probably due to the formation of shrinkage cracks reducing the tensile strength of the mortar.

Taking into consideration all the analysis presented above regarding the results of the presented tests, the following conclusions can be formulated:

- The presented research showed a high sensitivity of the tested mortar dedicated to bricklaying walls made of clinker and ceramic elements with an absorption capacity below 10% (low IRA brick) to the amount of mixing water used, which is very important when carrying out bricklaying in the summer period.
- The mortar, which was prepared with the amount of water recommended by the manufacturer, after 14 days of curing, achieved almost the maximum values of both tested parameters, i.e., flexural and compressive strengths.
- An increase in the amount of mixing water by about 6% and 18% in relation to the maximum value recommended by the manufacturer had such a negative impact on both analyzed mechanical parameters of the mortar that the mortar did not meet the requirements for the assumed strength class M10. In addition, with a larger amount of mixing water, the mortars reached the values of both strengths close to 28 days only after 21 days, which is a very bad situation in the case of using façade panels in the prefabrication, where a very important parameter is a rapid increase in load-bearing capacity in the initial period.
- The tests showed that in the case of the analyzed mortar, the maximum values of flexural and compressive strength were obtained for 21 days of curing. The samples tested after 28 days had slightly lower values. This also applied to the mortar made in accordance with the manufacturer’s recommendations, but in this case, only the compressive strength was lower by about 1% after 28 days than after 21 days.
- In the case of using more mixing water for mortar preparation, the decrease in the values of flexural strength after 28 days were 18% (for 6% more mixing water) and 26% (for 18% more water), respectively, and the compressive strengths were lower by 12% and 20%, respectively.
- The flexural strength to compressive strength ratio did not remain constant throughout the 28 day hardening period. For the mortar prepared in accordance with the manufacturer’s recommendations, after 14 days, the values were about 0.28–0.29, remaining at a constant level. Mortars made with more mixing water achieved lower values of this ratio, approximately 0.25–0.26, later, after 21 days of hardening.

Summarizing the above conclusions, it can be stated that great caution should be exercised when using this type of mortar dedicated to façade and facing walls in situations where it is necessary to use more mixing water than recommended by the mortar manufacturer.

## Figures and Tables

**Table 1:** Test program—preparation and conditions of storing specimens.

Mixing Water Content (Liters for 25 kg Bag)	Total Curing Time (Days)	No of Samples ^(1)^	Storage Time at a Temperature of 20 °C (±2 °C) (Days)		
			In the Mold	With the Mold Removed in Polyethylene Bag	In Humidity 65% ± 7%
4	5	3/6	3	2	-
	9	3/6	3	4	2
	14	3/6	3	4	7
	21	3/6	3	4	14
	28	3/6	3	4	21
4.5	5	3/6	3	2	-
	9	3/6	3	4	2
	14	3/6	3	4	7
	21	3/6	3	4	14
	28	3/6	3	4	21
5	5	3/6	3	2	-
	9	3/6	3	4	2
	14	3/6	3	4	7
	21	3/6	3	4	14
	28	3/6	3	4	21

^(1)^ Number of specimens for determination: flexural strength/compressive strength.

**Table 2:** Flexural and compressive strengths—mortar made with 4 L of mixing water per 25 kg bag.

Sample	Force (kN)		Flexural Tensile Strength (MPa)		Sample	Force (kN)		Compressive Strength (MPa)		CoV (%)
	F_max,i_	F_av_	f_mx_	f_mx,av_		F_c,i_	F_c,av_	f_c_	f_c,av_	ν_L_
**mFT-05_4L**	0.62	0.64	1.44	1.50	**mCT-05_4L**	12.83	12.36	8.02	7.72	4.9
						12.92		8.08		
	0.71		1.67			13.29		8.31		
						12.43		7.77		
	0.59		1.39			11.40		7.13		
						12.21		7.63		
**mFT-09_4L**	0.73	0.71	1.71	1.68	**mCT-09_4L**	13.31	14.76	8.32	9.22	10.2
						16.66		10.41		
	0.75		1.76			15.65		9.78		
						14.97		9.36		
	0.67		1.56			14.99		9.37		
						13.17		8.23		
**mFT-14_4L**	1.21	1.24	2.84	2.92	**mCT-14_4L**	16.23	16.01	10.14	10.01	3.1
						16.09		10.06		
	1.32		3.09			15.51		9.69		
						15.00		9.38		
	1.20		2.81			16.19		10.12		
						16.04		10.03		
**mFT-21_4L**	1.17	1.25	2.75	2.94	**mCT-21_4L**	17.34	17.35	10.84	10.84	7.1
						18.59		11.62		
	1.46		3.43			16.92		10.58		
						19.03		11.89		
	1.12		2.63			16.11		10.07		
						16.12		10.08		
**mFT-28_4L**	1.11	1.24	2.61	2.91	**mCT-28_4L**	15.04	16.69	9.40	10.43	7.0
						16.59		10.37		
	1.26		2.95			16.43		10.27		
						17.59		10.99		
	1.35		3.17			18.39		11.49		
						16.08		10.05		

**Table 3:** Flexural and compressive strengths—mortar made with 4.5 L of mixing water per 25 kg bag.

Sample	Force (kN)		Flexural Tensile Strength (MPa)		Sample	Force (kN)		Compressive Strength (MPa)		CoV (%)
	F_max,i_	F_av_	f_mx_	f_mx,av_		F_c,i_	F_c,av_	f_c_	f_c,av_	ν_L_
**mFT-05_4.5L**	0.58	0.58	1.36	1.36	**mCT-05_4.5L**	9.16	9.15	5.73	5.72	2.1
						8.91		5.57		
	0.68		1.59			7.84		4.90		
						9.05		5.66		
	0.49		1.14			9.21		5.76		
						9.42		5.89		
**mFT-09_4.5L**	0.82	0.71	1.92	1.67	**mCT-09_4.5L**	12.46	13.04	7,79	8.15	6.5
						11.85		7,41		
	0.69		1.62			13.76		8.60		
						8.75		5.47		
	0.63		1.47			13.40		8.38		
						13.73		8.58		
**mFT-14_4.5L**	0.79	0.75	1.85	1.5	**mCT-14_4.5L**	13.72	14.50	8.58	9.06	4.9
						14.04		8.78		
	0.83		1.3			14.16		8.85		
						14.37		8.98		
	0.63		1.47			15.52		9.70		
						15.18		9.49		
**mFT-21_4.5L**	1.17	1.09	2.74	2.56	**mCT-21_4.5L**	17.40	16.18	10.88	10.11	6.9
						17.52		10.95		
	1.00		2.35			16.01		10.01		
						16.24		10.15		
	1.11		2.61			14.86		9.29		
						15.05		9.41		
**mFT-28_4.5L**	1.04	1.02	2.45	2.39	**mCT-28_4.5L**	14.37	14.72	8.98	9.20	3.8
						14.37		8.98		
	1.06		2.48			14.23		8.89		
						14.49		9.06		
	0.95		2.23			15.57		9.73		
						15.28		9.55		

**Table 4:** Flexural and compressive strengths—mortar made with 5 L of mixing water per 25 kg bag.

Sample	Force (kN)		Flexural Tensile Strength (MPa)		Sample	Force (kN)		Compressive Strength (MPa)		CoV (%)
	F_max,i_	F_av_	f_mx_	f_mx,av_		F_c,i_	F_c,av_	f_c_	f_c,av_	ν_L_
**mFT-05_5L**	0.66	0.65	1.55	1.53	**mCT-05_5L**	8.93	8.56	5.58	5.35	5.4
						8.70		5.44		
	0.67		1.57			9.00		5.63		
						8.35		5.22		
	0.63		1.48			7.84		4.90		
						8.96		5.60		
**mFT-09_5L**	0.57	0.64	1.34	1.51	**mCT-09_5.L**	11.01	11.12	6.88	6.95	6.3
						9.97		6.23		
	0.68		1.59			11.81		7.38		
						11.07		6.92		
	0.68		1.59			11.29		7.06		
						11.53		7.21		
**mFT-14_5L**	0.82	0.75	1.92	1.76	**mCT-14_5L**	12.40	14.52	7.75	9.08	6.5
						14.08		8.80		
	0.66		1.55			14.76		9.23		
						15.09		9.43		
	0.77		1.80			14.31		8.94		
						14.38		8.99		
**mFT-21_5L**	1.12	0.96	2.63	2.25	**mCT-21_5L**	14.40	14.51	9.00	9.07	5.4
						15.54		9.71		
	0.92		2.16			13.59		8.49		
						14.85		9.28		
	0.84		1.97			13.62		8.51		
						15.06		9.41		
**mFT-28_5L**	1.08	0.92	2.53	2.15	**mCT-28_5L**	14.57	13.42	9.11	8.39	6.9
						14.24		8.90		
	0.89		2.09			13.71		8.57		
						12.14		7.59		
	0.78		1.83			12.65		7.91		
						13.20		8.25		

## Data Availability

Data available on request.
